# Assessing the Impact of Social and Psychological Factors on Consumers’ Willingness to Pay for Low-Carbon Beef: Evidence from Urban China

**DOI:** 10.3390/foods15061023

**Published:** 2026-03-15

**Authors:** Jiajie Li, Yingying Lin, Xinyu Bai

**Affiliations:** School of Agricultural Economics and Rural Development, Renmin University of China, Beijing 100872, China; lyy18801050145@163.com (Y.L.); 18563508869@163.com (X.B.)

**Keywords:** low-carbon beef, willingness to pay, warm glow feelings, protest beliefs, social norms, contingent valuation, inferred valuation, China

## Abstract

Reducing anthropogenic greenhouse gas (GHG) emissions across beef production raises critical questions about consumers’ acceptance and willingness to pay (WTP) for low-carbon beef. As a purely environmental attribute, low-carbon choices are often driven by social and psychological motivations rather than direct personal benefit. This study aims to identify how the social and psychological factors of warm glow feelings, protest beliefs, and social norms influence Chinese urban consumers’ WTP for low-carbon beef. Utilizing survey data from 760 consumers in Beijing, we employed both the double-bounded dichotomous choice contingent valuation method (CVM) and the inferred valuation method (IVM) to assess consumers’ own WTP and inferred WTP for low-carbon beef. The results showed that urban Chinese consumers generally indicated a willingness to pay a premium for low-carbon beef with mean own and inferred WTP values at RMB 47 and RMB 45.29 per 500 g, representing premium rates of 17.49% and 13.23%, respectively. Consumers’ warm glow feelings, protest beliefs, and social norms significantly influenced their own WTP for low-carbon beef, whereas their inferred WTP was mainly affected by social norms. Consumers’ environmental concern had no statistically significant effect on either own WTP or inferred WTP. Policymakers should frame low-carbon beef consumption as a source of personal psychological benefit, mandate transparency regarding the allocation of premium payments of low-carbon beef and establish low-carbon consumption role models within communities.

## 1. Introduction

One of the major drivers of climate change, the global food system is estimated to be responsible for up to 30% of anthropogenic greenhouse gas (GHG) emissions [1,2,3], with animal-based production being one of the main contributors [1,4]. In particular, beef production accounts for 32% of global GHG emissions from animal-based emissions [5]. Beyond reducing consumer demand for beef, lowering emissions in the beef production process may be the most viable solution for achieving climate goals [6]. Strategies and technologies, such as developing novel feed additives that reduce methane emissions during cattle digestion, as well as enhancing carbon sequestration through improved grazing management and the planting of legumes on pastures, have been actively pursued worldwide to mitigate the environmental impact of beef production [7]. However, GHG emission reduction measures across the entire beef production system may lead to price increases for the final product due to rising costs, raising critical questions about consumers’ acceptance and willingness to pay (WTP). Beef producers will only be incentivized to adopt long-term implementation of GHG mitigation if consumers are willing to pay a price premium for low-carbon beef [8]. Therefore, understanding consumer-driven, bottom-up demand for low-carbon beef has become crucial for reforming the beef production system.

Many empirical studies have investigated the factors that influence the willingness of consumers to buy environmentally friendly foods. The major factors include environmental concern [9], knowledge and information provision [6,10,11], value and belief [12,13,14], life styles and habit [15], and socio-demographic factors [16]. However, the fact that pro-environmental behavior is shaped by social and psychological factors remains unaccounted for in predicting actual sustainable behavior [17]. In particular, unlike impure environmental attributes (organic, all natural, local, grass-fed, etc.), which offer personal health or safety benefits alongside environmental protection, low-carbon is a pure environmental attribute [8]. For purely environmental attributes, social and psychological factors may prevail in shaping consumer behavior, as the underlying motivation stems from altruistic or social motives rather than direct personal benefit [18,19]. If people experience “warm glow” emotions, these psychological benefit may motivate them to engage in pro-environmental behavior [20]. Conversely, when people hold “protest beliefs” about an environmental initiative, they are likely to respond with resistance to the process [21]. In the literature on consumer pro-environmental behavior, the effects of warm glow feelings and protest beliefs on consumers’ willingness to pay for environmentally friendly products have typically been examined separately. However, the psychological mechanisms, particularly in terms of the role of positive warm glow feelings and negative protest beliefs, remain under-examined in the context of environment-friendly food consumption. Additionally, according to focus theory of normative conduct, social norms as external factors have been proven to be a powerful influence on individual pro-environmental intentions and behaviors [22,23,24,25]. However, few studies have integrated both internal psychological factors (warm glow feelings and protest beliefs) and external social factors (social norms) into a single theoretical framework to explore their joint impact on consumers’ WTP on environmentally friendly foods [26]. This research gap emphasizes the need for examining the social and psychological variables together to better understand consumers’ decision-making process in relation to environmentally friendly foods. Accordingly, this study assesses how three social and psychological factors, namely, warm glow feelings, protest beliefs, and social norms, along with their interaction effects, influenced consumers’ WTP for low-carbon beef.

Extensive research has explored consumers’ acceptance of eco-labeled foods in developed countries, but studies on willingness to pay for low-carbon foods in developing countries, particularly China, are limited [27,28,29]. As the country with the largest carbon emissions and second-largest beef consumption, China faces immense pressure to reduce GHG emissions of beef production. It is necessary to understand Chinese consumers’ intention and demand for low-carbon beef. Thus, this study selected Chinese consumers as the research subjects. Specifically, our study focused on urban consumers in China because, according to the China Statistical Yearbook, these consumers usually consume twice the amount of beef products, and the environmental impact is higher than that of rural consumers.

Accordingly, we aim to answer two primary research questions: (1) How do the social and psychological factors of warm glow feelings, protest beliefs, and social norms, along with their interaction effects, influence Chinese urban consumers’ WTP for low-carbon beef? (2) What are the prices that Chinese urban consumers are willing to pay for low-carbon beef?

The significance of this study lies in its incorporation of warm glow theory and focus theory of normative conduct to explore the effects of warm glow feelings, protest beliefs, and social norms on consumers’ WTP for low-carbon foods. This research contributes to our knowledge of food system transformation by emphasizing the role of consumer psychology and sociology in predicting consumers’ behavioral responses to low-carbon foods. These insights could help policymakers tailor their strategies to promote the widespread societal adoption of low-carbon food consumption and achieve a sustainable transformation of the food system.

## 2. Literature Review

### 2.1. Consumers’ WTP for Beef with Low-Carbon Attributes

Several studies have examined consumers’ WTP for beef with low-carbon attributes. Most studies have found that consumers are willing to pay a positive premium [6,12,30]. Yang and Renwick [31] conducted a meta-analysis based on 94 studies globally and found that consumers are willing to pay an 18.9% premium for beef with environmentally friendly attributes. However, there is notable heterogeneity in WTP across populations from different countries and regions. For instance, White and Brady [8] predicted that North American consumers would be willing to pay an average premium of 14.8% for beef with pure environmental attributes. Li, Jensen, Clark and Lambert [9] reported that the average U.S. household is willing to pay a 10% price premium for “Raised Carbon Friendly”-certified beef. Xu and Lin [32] reported that Chinese consumers were willing to pay 8.43% more than the original price for carbon-labeled food. Charry et al. [33] found that the price premiums of consumers in Colombia is 23.17% for beef with an eco-friendly certification relative to conventional beef. Payen et al. [34] reported that European consumers are willing to pay a 32% price premium on average, ranging from 18% to 103%, for low environmental footprint beef. Chen, Zhen, Li, Yang and Ren [27] found that Chinese urban consumers even had a RMB 28.92/500 g price premium with a premium rate over 50% for carbon-neutral-labeled beef. However, studies have also found that consumers exhibit a negative willingness to pay for low-carbon beef. For instance, De Valck et al. [35] reported that Australian consumers are unwilling to pay a premium for beef produced with lower GHG emissions. Lami et al. [36] also demonstrated that Spanish consumers show negative willingness to pay for beef with carbon footprint labeling. The substantial variation in WTP across consumer groups underscores the critical importance of investigating the determinants of willingness to pay for low-carbon beef.

### 2.2. Impact of Warm Glow Feelings, Protest Beliefs, and Social Norms on Consumers’ WTP for Pro-Environmental Food

Andreoni [19,20] argued that consumers can derive “warm glow” utility from the contribution to the public good. Besides altruism, participation in sustainable action also yields a psychological benefit of moral satisfaction and motivates further prosocial behavior [21]. Warm glow feelings have been widely reported to positively influence consumers’ pro-environmental behavior [18,20,37,38]. Only recently have researchers begun to explore how the intrinsic rewards of warm glow feelings affect consumer purchase intentions for pro-environmental foods. Cahyasita et al. [39], Boobalan, Nachimuthu and Sivakumaran [26], and Wang et al. [40] have reported that the intrinsic rewards of warm glow feelings have led to consumers’ positive purchase intention regarding organic foods and green foods. However, Iweala et al. [41] found that the effect of warm glow feelings on consumers’ purchase intention for foods with a carbon-neutral claim depended critically on their level of label awareness. This suggests that it is necessary to explore the influence of warm glow feelings on consumers’ WTP for low-carbon food.

Protest beliefs refer to another psychological factor in willingness to pay surveys where respondents reveal a protest attitude toward the evaluation process [21]. Protest beliefs manifest in various forms, for example, questioning the fairness of payment, doubting the cash-based payment method, and worrying about free-riding [37]. Protest beliefs have been examined as determinants that shape consumers’ WTP during the environmental valuation [42,43]. Despite extensive research, the direction of the impact of protest beliefs on consumers’ WTP remains controversial. For instance, Jorgensen and Syme [21] and Meyerhoff and Liebe [43] reported that the level of protest beliefs had a negative impact on consumers’ WTP for environmental abatement. However, Grammatikopoulou and Olsen [37] argued that respondents holding protest beliefs had a higher average willingness to pay for an environmental protection program. Lo and Jim [42] demonstrated the existence of both positive and negative relationships between protest beliefs and consumers’ WTP for urban tree protection. Protest beliefs regarding the valuation method and fairness decreased respondents’ willingness to pay. In contrast, protest beliefs concerning a hypothetical scenario showed a significant positive correlation with their willingness to pay. Therefore, the complex relationship between protest beliefs and willingness to pay requires a more detailed analysis of respondents’ specific protest items.

Perkins and Berkowitz [44] conceptualized social norms as attitudes perceived to be held by other people. Cialdini, Reno and Kallgren [25] further divided social norms into descriptive norms and injunctive norms. Descriptive norms refer to prevalence of a behavior among other people, and injunctive norms are concerned with the approval of a behavior by other people [45]. Descriptive norms and injunctive norms have disparate impacts on human behavior. For example, de Leeuw et al. [46] found that only descriptive norms independently predicted behavioral intentions of high-school students, but research on how descriptive norms and injunctive norms separately influence consumers’ acceptance of low-carbon foods remains limited [47]. Additionally, Muthukrishna and Schaller [48] have pointed out that the role of social norms is high for collectivistic cultures compared with individualistic cultures. Therefore, it is particularly necessary to study the influence of social norms within China’s unique cultural contexts.

## 3. Methods

### 3.1. Contingent Valuation and Inferred Valuation Experimental Design

The contingent valuation method (CVM) is an important stated preference method in non-market value evaluation [49]. However, research has found that people usually overstate their preferences in a hypothetical scenario due to the psychological utility or social desirability bias [50]. Lusk and Norwood [51] have proposed the inferred valuation method (IVM) by asking respondents to predict the WTP of general consumers to mitigate the hypothetic bias. The IVM has been used for evaluating environmental goods or services [52,53] and foods with climate-neutral labels [54]. Therefore, this study adopted both the CVM and IVM to evaluate consumers’ actual WTP for low-carbon beef.

We first used the double-bounded dichotomous choice contingent valuation method (DBDC-CVM) to estimate consumers’ own WTP for low-carbon beef [55]. Before the survey, we conducted a market survey on the price of raw beef in Beijing and found that the average price of raw beef was RMB 40/500 g. According to White and Brady [8], who reported that North American consumers would pay a 6.7% to 32.6% premium for beef with pure environmental labeling, in this study, we justified a 5–30% premium as our initial bidding range. Six first bidding prices B_0_ were set by increasing RMB 40/500 g by 5%, 10%, 15%, 20%, 25%, and 30%, and ultimately forming six levels of RMB 42, 44, 46, 48, 50, and 52/500 g, respectively. The second bidding prices were set with RMB 2/500 g higher (B_H_) or lower (B_L_) than the first bidding price in each bid scenario, except in the first scenario where B_L_ is RMB 1/500 g lower than first bidding price, indicating a very low willingness to pay a premium for low-carbon beef.

Because respondents may not be aware of low-carbon foods, a statement providing basic information on the definition of such foods and pictorial examples of such food products available on the market was provided to each respondent prior to their exposure to the questions. Then, each respondent was asked to read a cheap talk script described as “Imagine you are shopping in a real supermarket where you need to buy fresh raw beef. The price of conventional beef is RMB 40/500 g. A new type of beef, known as low-carbon beef, has also appeared in the supermarket. It exhibits no differences in nutrition, taste, or appearance compared with conventional beef, but it generates lower carbon emissions during the production process”. Respondents were then randomly assigned one of the bid scenarios and asked if they were willing to pay the B_0_ price for low-carbon beef. If respondents answered “Yes”, they would be asked a higher bid quote of B_H_ as the second question; otherwise, they would be provided a lower bid quote of B_L_. In addition to measuring respondents’ own WTP, we applied the IVM to measure their inferred WTP. Respondents would be continually asked if an ordinary person would be willing to pay the B_0_ price for low-carbon beef on average. If respondents answered “Yes”, they would be asked a higher bid quote of B_H_ as the second question; otherwise, they would be provided a lower bid quote of B_L_. The questionnaire is presented in the Supplementary Materials (File S1).

### 3.2. Measurement

We measured individuals’ levels of warm glow feelings, protest beliefs, social norms, and environmental concern using psychometric scales. A psychometric scale is a type of psychological measurement involving asking people to answer Likert-type scale questions wherein they indicate their level of agreement with a list of statements from 1 (strongly disagree) to 5 (strongly agree). Higher scores reflected a stronger sense of agreement.

Warm glow feelings: We designed four items adapted from Hartmann, Eisend, Apaolaza and D’Souza [18], Iweala, Spiller and Meyerding [41], and van der Linden [20] to measure respondents’ warm glow feelings. The four items covered different facets of warm glow motivation: self-confirmation (e.g., “WG1: I would feel good about myself if I decided to take personal action to help reduce climate change”), altruistic affect (e.g., “WG2: I would feel positive if I contribute to the well-being of humanity and nature”), social recognition (e.g., “WG3: Buying low-carbon foods makes me feel respected”), and personal emotional satisfaction related to low-carbon consumption (e.g., “WG4: Buying low-carbon foods gives me a pleasant feeling of personal satisfaction”).

Protest beliefs: Based on the research of Jorgensen and Syme [21], Meyerhoff and Liebe [43], and Lo and Jim [42], we developed five items to measure respondents’ protest beliefs. The items collectively captured resistance to personal payment (e.g., “PB1: I refuse to contribute to carbon reduction in monetary terms”), perceptions of unfairness (e.g., “PB2: It is unfair for me to pay more money for low-carbon foods”), doubt regarding the legitimacy of responsible entities (e.g., “PB3: The government should pay to reduce carbon emissions from foods” and “PB4: Those food companies with high carbon emissions should pay for the measures”), and concerns about low social participation (e.g., “PB5: My money won’t make a difference because most people are not willing to pay for it”).

Social norms: Based on the design of Boobalan, Nawaz, R. M. and Gajenderan [47], we developed two items standing for each type of social norm. For descriptive norms, we designed the statement, “SN1: My family and friends would prefer to pay more for low-carbon foods”, to capture perceptions of what people with close relationships with the participant typically do. For injunctive norms, we designed the statement, “SN2: People around me generally believe that buying low-carbon foods is more beneficial for the environment”, to capture perceptions of socially approved and disapproved behaviors [56].

Environmental concern: Concern about environmental problems was measured with four items. The scale conceptualizes environmental concern as a broad cognitive factor that measures individuals’ awareness of environmental issues (e.g., “Environmental issues are more critical now than in the past decade”), the specific issue of climate change (e.g., “The current global situation of greenhouse gas emissions is quite severe”), and perceived severity of risks both in the present (e.g., “Climate change has already affected my daily life”) and future (e.g., “If current trends continue, we will soon suffer a severe environmental disaster”).

### 3.3. Data Collection

This study selects urban consumers in Beijing as the research subjects because low-carbon food is still a novel concept in China, and products bearing low-carbon labels are primarily available in major cities such as Beijing. Urban consumers in the Beijing market are more likely to encounter low-carbon food and have the opportunity to make choices regarding it. Based on preliminary research, the formal survey was conducted in May 2024, utilizing a combination of online and offline methods. The offline surveys were conducted through random face-to-face interviews at shopping malls in Beijing. Online surveys were distributed to consumers in Beijing via Sojump (Changsha Ranxing Information Technology Co., Ltd., Changsha, China), a leading data collection service provider in China. The sample size was estimated a priori using G*Power 3.1. A two-tailed test was specified with an odds ratio of 1.5 (small to medium effect size), a baseline probability Pr = 0.2, a significance level α = 0.05, and a statistical power of 1 − β = 0.95. The calculated minimum required sample size was 503. Considering an anticipated 20% rate of invalid responses, the final target was to collect at least 604 valid questionnaires. Our data collection resulted in 816 questionnaires. To address the limitations of online surveys, such as the absence of face-to-face interaction, this study implemented the following control measures to ensure the reliability and quality of the data. Firstly, strict screening of respondents was conducted. We ensured that the surveyed respondents were residents of Beijing by restricting the IP addresses and including a geographic location question. Secondly, the reliability of the survey was strictly controlled. We included a mandatory question in the questionnaire, and surveys that failed to provide an accurate answer to this question were excluded. Additionally, questionnaires with suspiciously short response times were excluded. This ultimately resulted in 760 valid questionnaires, yielding a valid response rate of 93.1% and satisfying the a priori sample size requirement. Of these, 150 were collected from offline surveys and 610 from online surveys. Homogeneity tests showed no significant differences between the online and face-to-face respondents regarding gender (χ^2^ = 3.526, *p* = 0.060), monthly income (χ^2^ = 10.037, *p* = 0.074), religion (χ^2^ = 0.688, *p* = 0.407), and beef consumption frequency (χ^2^ = 4.068, *p* = 0.540). Although statistically significant differences (*p* < 0.05) were found between the two groups for the variables of age, education level, and presence of children in the household, the substantial disparity in group sizes indicated that relying solely on *p*-values could lead to overinterpreting minor differences. Therefore, we further calculated their effect sizes and the results showed that Cramér’s V values for these three variables ranged from 0.126 to 0.173, all falling within the small effect range. This indicated that the differences were practically insignificant and the samples obtained from the two channels were generally homogeneous. Therefore, we used the combined sample for the subsequent statistical analyses.

Table 1 lists the summary statistics of demographic variables. The full sample comprised 409 females and 351 male respondents, suggesting a balanced number of women and men. Regarding age, 78.03% of the respondents were 18–35 years old. Regarding education, respondents with a bachelor’s degree accounted for 90.13% of the total sample. Respondents’ monthly incomes were almost evenly distributed between RMB 3000 and 20,000. Moreover, 27.63% of the respondents had children under 12 years old in their families and only 4.61% of the respondents had a religion. Finally, 41.19% of the respondents ate beef 2–3 times per month and 35.26% of the respondents had an even higher frequency of beef consumption.

### 3.4. Empirical Specification

Following the approach of Cranfield [57] and Zhang et al. [58], we expressed respondent i’s own WTP as follows:

(1) $${WTP}_{i}=x_{i}^{\prime }\beta +\mu_{i}$$

where $x_{i}^{\prime }$ is a vector of respondent characteristics including a set of variables that measure the respondent’s warm glow feelings, protest beliefs, social norms, environmental concern, and demographic variables; β is the corresponding coefficient vector; and $\mu_{i}$ is an error term that is normally distributed with mean zero and finite variance $\sigma^{2}$.

A respondent’s responses to the first and second bid questions could form four possible patterns, which were “Yes-Yes”, “Yes-No”, “No-Yes”, and “No-No”. We named them as binary valued indicators $d_{i}^{yy}$, $d_{i}^{yn}$, $d_{i}^{ny}$, and $d_{i}^{nn}$ according to consumer i’s choice. Each respondent’s response can be considered as a function of true WTP, which is a random variable with a cumulative distribution function defined as $\Phi \left(\cdot \right)$. The probabilities of four possible response patterns can be expressed as follows:

(2) $$Pr\left(d_{i}^{yy}=1|x_{i}\right)=\mathrm{Pr}({WTP}_{i}\geq B_{iH})=1-\Phi \left(\frac{B_{iH}-x_{i}\beta }{\sigma }\right)$$

(3) $$Pr\left(d_{i}^{yn}=1|x_{i}\right)=\mathrm{Pr}({B_{io}\leq WTP}_{i}<B_{iH})=\Phi \left(\frac{B_{iH}-x_{i}\beta }{\sigma }\right)-\Phi \left(\frac{B_{iO}-x_{i}\beta }{\sigma }\right)$$

(4) $$Pr\left(d_{i}^{ny}=1|x_{i}\right)=\mathrm{Pr}({B_{iL}\leq WTP}_{i}<B_{iO})=\Phi \left(\frac{B_{iO}-x_{i}\beta }{\sigma }\right)-\Phi \left(\frac{B_{iL}-x_{i}\beta }{\sigma }\right)$$

(5) $$Pr\left(d_{i}^{nn}=1|x_{i}\right)=\mathrm{Pr}({WTP}_{i}<B_{iL})=\Phi \left(\frac{B_{iL}-x_{i}\beta }{\sigma }\right)$$

where β and σ can be estimated by maximizing the log-likelihood function:

(6) $$\ln L\left(\beta ,\sigma \right)=\sum_{i=1}^{N}\left\{d_{i}^{yy}\ln\left[1-\Phi (\frac{B_{iH}-x_{i}\beta }{\sigma })\right]+d_{i}^{yn}\ln\left[\Phi \left(\frac{B_{iH}-x_{i}\beta }{\sigma }\right)-\Phi \left(\frac{B_{iO}-x_{i}\beta }{\sigma }\right)\right]+d_{i}^{ny}\ln\left[\Phi \left(\frac{B_{iO}-x_{i}\beta }{\sigma }\right)-\Phi \left(\frac{B_{iL}-x_{i}\beta }{\sigma }\right)\right]+d_{i}^{nn}\ln\left[\Phi \left(\frac{B_{iL}-x_{i}\beta }{\sigma }\right)\right]\right\}$$

The model was implemented using the doubleb command in Stata 16.0. The maximum likelihood estimation value of parameter β allows for the calculation of WTP in Equation (1). We calculated respondent i’s inferred WTP with the same method.

## 4. Results

### 4.1. Sample Characteristics

Table 1 and Table 2 display the descriptive statistics of the demographic variables. Table 3 shows that all measurement scales demonstrated good internal consistency (with Cronbach’s α values > 0.7). We conducted principal component analysis (PCA) separately on the items for warm glow feelings, protest beliefs, social norms, and environmental concern. In each case, the PCA yielded a single reliable component with a variance explanation rate exceeding 60%, which indicates a satisfactory level of explanatory power. Bartlett’s test of sphericity was significant (*p* < 0.001) for all scales, supporting factorability. For the two-item social norms scale, the KMO measure is less informative. Validity was instead confirmed with a strong inter-item correlation (r = 0.634, *p* < 0.001) and high factor loadings, justifying the creation of a composite score.

The response distribution in six bid scenarios for the DBDC-CVM and IVM is illustrated in Table 4. Overall, the sample sizes in the six bid scenarios were well balanced. In both CVM and IVM surveys, the proportion of respondents answering “yes-yes” consistently decreased as the initial bid amount increased. This pattern indicates that the willingness to purchase low-carbon beef declined with higher prices, which aligns with general economic expectations. Across all bid scenarios, the proportion of respondents who stated they would accept a given bid price themselves was consistently higher than the proportion who inferred that the general public would accept it. This result clearly demonstrates a divergence between own WTP and inferred WTP.

### 4.2. Estimation Results of the Own WTP Model

Table 5 presents the maximum likelihood estimates of respondents’ own WTP for low-carbon beef. Model 1 did not include any explanatory variables. The constant term indicated that without considering the influence of any factors, the respondents’ average willingness to pay was RMB 47.012/500 g, which was higher than the average price of conventional raw beef at RMB 40/500 g. This suggested that the respondents were willing to pay a premium for low-carbon beef. Model 2 incorporated the four variables of warm glow feelings, protest beliefs, social norms and environmental concern. The results showed that both warm glow feelings and social norms had a significant positive impact on respondents’ own WTP at the 1% level, whereas protest beliefs significantly negatively affected respondents’ own WTP at the 5% level. Respondents’ environmental concern had no significant impact on their own WTP. Model 3 further incorporated demographic control variables. The model’s McFadden’s pseudo-R^2^ was calculated as 0.143, which fell within the acceptable range for discrete choice models. Furthermore, the model’s overall Wald chi-square test was significant at the 1% level (*p* < 0.01), indicating that the selected variables have significant explanatory power for respondents’ willingness to pay. The results showed that warm glow feelings and social norms remained significantly positive, while protest beliefs remained significantly negative. This indicated that the influence of these factors on respondents’ own WTP was robust. Regarding respondents’ demographic variables, the presence of children in the household and a high frequency of beef consumption both had a significant positive impact on respondents’ own WTP for low-carbon beef at the 1% level. Respondents’ income status had a significantly positive impact on WTP at the 10% level. However, the level of education of the respondents showed a significantly negative impact on their WTP at the 1% level.

**Figure 1:**
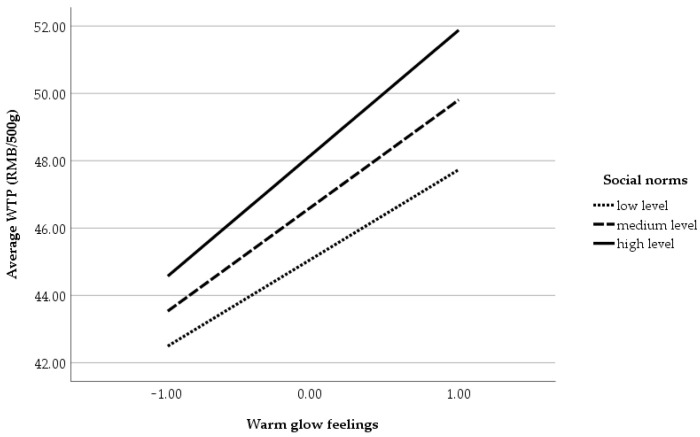
Interaction effect between warm glow feelings and social norms.

Table 6 presents the estimation results of interaction terms between variables. Model 4 illustrated the interaction effects between respondents’ warm glow feelings and their protest beliefs, social norms, and environmental concern. The results showed that only the interaction effect between warm glow feelings and social norms was significantly positive at the 5% level. As shown in Figure 1, when respondents perceived strong positive social norms associated with purchasing low-carbon beef, their sense of warm glow was further amplified and led to a significant increase in their willingness to pay a price premium. Model 5 further examined the interaction effects between respondents’ protest beliefs and social norms, as well as environmental concern. The results showed that none of the interaction terms were statistically significant. This may indicate that respondents’ protest beliefs exhibited relative stability in decision-making and were not easily altered by external social factors or general environmental attitudes.

We further estimated the models by replacing the composite variables of warm glow feelings, protest beliefs, and social norms with their original measuring items, with the results presented in Table 7. In Model 6, we substituted the variable of warm glow feelings with the original four items and the results showed that only WG1 had a significant positive impact on respondents’ own WTP. This indicated that not all positive and warm glow feelings can be effectively converted into WTP. Only the sense of self-affirmation derived from the specific consumption behavior itself significantly enhanced the willingness to pay for low-carbon beef, whereas broad altruistic affects or the respect and satisfaction gained from others did not significantly affect the premium. In Model 7, we replaced the variable of protest beliefs with the original five items and the results showed that the impact of different protest belief items on respondents’ WTP varied. Protest beliefs indicating that respondents opposed contributing to carbon emission reduction through monetary payment had a significantly negative impact on their own WTP at the 1% level. Interestingly, protest beliefs questioning the fairness of the payment method had a significantly positive impact on WTP at the 5% level. Protest beliefs of doubts regarding the legitimacy of responsible entities and concerns about low social participation showed no significant impact on respondents’ own WTP. In Model 8, we replaced the social norms variable with the descriptive norms item and the injunctive norms item. The results indicated that only descriptive social norms, especially proximal peer descriptive norms, exerted a significant positive effect on respondents’ willingness to pay. Injunctive social norms did not significantly affect own WTP.

### 4.3. Estimation Results of the Inferred WTP Model

The estimation results of the inferred WTP for low-carbon beef are presented in Table 8. Model 9 did not include any explanatory variables. The constant term indicated that without considering the influence of any factors, the respondents believed that the general public’s willingness to pay was RMB 45.392/500 g, which was lower than the own WTP in Model 1 but still higher than the average price of conventional raw beef at RMB 40/500 g. This suggested that the respondents inferred that general consumers would also pay a premium for low-carbon beef. Model 10 incorporated the four variables of warm glow feelings, protest beliefs, social norms and environmental concern. The results showed that only social norms had a significant positive impact on the inferred WTP at the 1% level, while warm glow feelings only positively affected the inferred WTP at the 10% level. Respondents’ protest beliefs and environmental concern had no significant impact on the inferred WTP. Model 11 further incorporated demographic control variables. The model’s McFadden’s pseudo-R^2^ was 0.067, which fell within the normal range for discrete choice models based on cross-sectional survey data. More importantly, the model’s overall Wald chi-square test was significant at the 1% level (*p* < 0.01), indicating that the included independent variables jointly had a statistically significant explanatory power for respondents’ willingness to pay. The results remained consistent with those of Model 10, indicating the impact of the key factors on inferred WTP was robust. Regarding the demographic variables of respondents, a high frequency of beef consumption still had a significantly positive effect on inferred WTP, while respondents’ education level remained significantly negatively associated with the inferred WTP at the 1% level.

### 4.4. WTP Analysis

**Figure 2:**
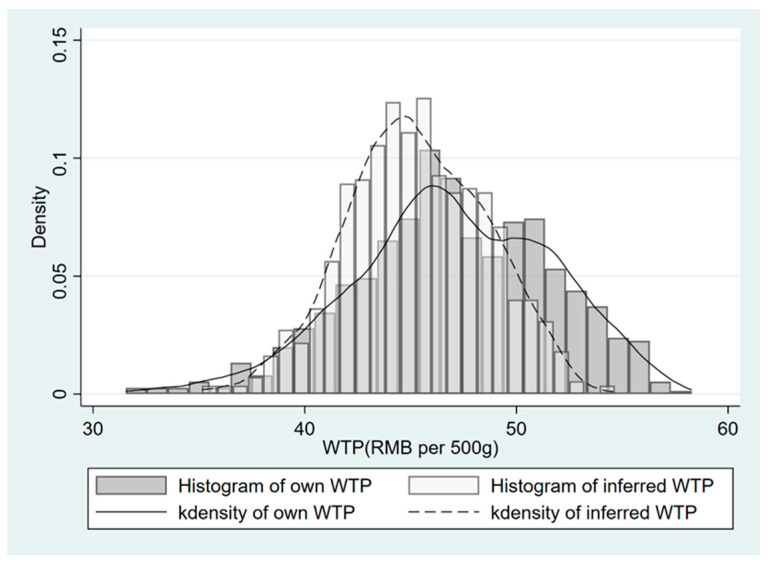
Histogram and density plots of own WTP and inferred WTP for low-carbon beef across the full sample.

Using the parameters estimated from WTP Models 3 and 11, we calculated the own WTP and inferred WTP for low-carbon beef across each respondent in our sample, and the descriptive statistics are presented in Table 9. Figure 2 presents the histogram and density plots for own WTP and inferred WTP for low-carbon beef. As expected, respondents’ average own WTP for low-carbon beef was RMB 47/500 g with a premium rate of 17.49%, while their average inferred WTP was RMB 45.29/500 g with a premium rate of 13.23%, resulting in a mean WTP gap of RMB 1.70/500 g. The results from a Wilcoxon signed-rank test indicated a statistically significant difference between own WTP and inferred WTP, with own WTP being significantly higher (Z = 18.56, *p* < 0.001, r = 0.67).

## 5. Discussion

The aim of this study was to examine the own WTP and inferred WTP for low-carbon beef and the factors that influenced them among Chinese urban consumers. Specifically, we explored how social and psychological factors of warm glow feelings, protest beliefs, and social norms influenced Chinese urban consumers’ WTP.

Our results demonstrated that warm glow feelings had a significantly positive influence on respondents’ own WTP. According to the warm glow of giving theory [59], humans gain utility from the act of giving. The warm glow feelings and happiness derived from giving are an innate driver that motivates individuals to engage in prosocial and pro-environmental activities such as accepting and paying a price premium for low-carbon beef. Our results were consistent with the findings of Taufik et al. [60], Hartmann, Eisend, Apaolaza and D’Souza [18], and van der Linden [20]. Regarding the predictive strength, warm glow feelings exerted the strongest effect, followed by social norms and then protest beliefs (in the negative direction). Our results are in line with Bergquist et al. [61]. Furthermore, our research showed that not all warm glow feelings significantly enhanced respondents’ WTP and only the sense of self-affirmation derived from the specific consumption behavior did. This indicated that the sense of respect or personal satisfaction derived from altruistic behavior was not the primary driver for purchasing low-carbon beef. Instead, willingness to pay increased only when respondents’ sense of self-efficacy and responsibility was activated, thereby confirming their identity as proactive agents in mitigating climate change [26].

In this study, we investigated whether the protest beliefs would affect the consumers’ own WTP for low-carbon beef. The results confirmed that respondents’ protest beliefs significantly and negatively affected their own WTP at the 5% level. Our further analysis revealed that protest beliefs were not a unidimensional variable. Different dimensions for protest beliefs exerted statistically divergent impacts. More specifically, respondents were strongly opposed to the idea of supporting carbon emission reduction through monetary payments and refused to pay a premium. This suggested that to encourage consumers to pay a premium for low-carbon food, it is essential to transparently disclose the use of the premium portion and clarify the connection between consumers paying a higher price and producers reducing carbon emissions. Unexpectedly, our results showed that the protest beliefs questioning fairness had a significantly positive effect on WTP at the 5% level. The positive relationship between protest beliefs and WTP has been found and discussed in the literature [42,43,62]. Frey and Pirscher [62] argued that protest responses should be viewed as expressions of respondents’ moral and social attitudes, rather than merely reflections of economic preferences. Therefore, in our research, respondents’ skepticism regarding payment fairness may also serve merely to express doubt about where primary responsibility should lie, but this did not indicate an unwillingness to pay a premium.

Our study showed the positive impact of social norms on respondents’ own WTP. When comparing the injunctive and descriptive norms between respondents, we found that descriptive norms exerted a significant positive effect on respondents’ willingness to pay, which is consistent with previous studies [22,23,28,46]. Our research confirmed that in a predominantly collectivist cultural context such as China, peer effects played an important role in predicting and influencing public environmentally responsible behavior.

In addition, we found that respondents’ environmental concern had no significant impact on their own WTP. Previous research findings on the relationship between environmental concern and sustainable consumption have been contentious [63]. Although some studies have reported that a high level of concern regarding environmental conditions led to a positive attitude toward low-carbon consumption [9,24,64,65], there are also many studies that suggest no inherent correlation between consumers’ environmental concern and sustainable consumption behavior [29,66,67]. Our findings confirmed that enhancing consumers’ environmental concern may not be the pivotal factor in promoting their willingness to attribute a positive price premium to low-carbon products. The attitude-behavior gap suggests that only a small fraction of pro-environmental behavior can be directly linked to environmental concern [68]. Internal factors of motivation, emotional involvement, and responsibility, along with external factors such as social norms, have a greater influence on pro-environmental behavior [69,70].

From the perspective of the interaction between variables, our analysis further complemented the impact mechanism of the variables. First, there was no significant moderating effect of protest beliefs on the relationship between warm glow feelings and WTP, indicating that the positive effect of warm glow feelings remained robust regardless of consumers’ protest beliefs. Second, social norms not only positively influenced consumers’ own willingness to pay for low-carbon beef, but also further enhanced this willingness by significantly eliciting a warm glow effect among consumers. Our interaction effect is in line with Halvorsen [71] and Abbott et al. [72], highlighting the critical importance of social norms [24]. Third, the interaction effects between environmental concerns and both warm glow feelings and protest beliefs were found to be statistically non-significant. This suggested that the influence of warm glow feelings or protest beliefs on consumers’ own WTP operated independently of the level of environmental concern among consumers. Our findings imply that in urban China, the emotional variables of warm glow feelings and protest beliefs and the contextual factors of social norms played more decisive roles than the attitudinal variable to predict consumers’ own WTP for low-carbon beef.

Beyond measuring an individual’s own WTP, we also measured the inferred WTP by asking for respondents’ forecasts of others’ choices. These forecasts served as a more accurate proxy for actual valuation [73]. As expected, the results showed that our samples’ mean inferred WTP (RMB 45.29/500 g) was significantly lower than the mean own WTP (RMB 47/500 g), which is in line with previous studies of inferred valuation estimates [52,53,74]. The results of the maximum likelihood estimate of the inferred WTP showed that the factors of warm glow feelings and protest beliefs, which had a statistically significant effect on own WTP, exhibited a markedly diminished impact on inferred WTP. This indicated that the WTP gap between own WTP and inferred WTP could be due to moral or emotional motives [53]. Notably, even after adjustment, the inferred WTP demonstrated a persistent average premium of RMB 5.29/500 g for low-carbon beef over conventional beef priced at RMB 40/500 g, which represented a premium rate of 13.23%. This indicated that urban Chinese consumers maintained a willingness to pay a price premium for low-carbon beef. Our findings on the premium rate for low-carbon beef fell within the 10–18.9% range documented in previous global studies on beef with pro-environmental attributes [8,9,31]. We also compared our results with those from recent studies on Chinese consumers’ willingness to pay for environmentally friendly foods. Our estimated premium rate was comparable to that of Xu and Lin [32], but generally lower than the rates (26% and above) reported in other studies [27,29,50,75,76,77,78]. There are two possible reasons for this discrepancy. First, most of those studies used choice experiments to estimate willingness to pay [27,50,76,78], and previous research has shown that premium rates derived from choice experiments tend to be higher than those from contingent valuation methods [79,80]. Second, the eco-labels in some of these studies [75,77] involved impure environmental attributes, which may have led consumers to account for personal benefits when making valuations. Our research identified that social norms significantly positively affected the inferred WTP, which provided further evidence for the critical role of social norms in shaping actual WTP for low-carbon beef among Chinese urban residents.

## 6. Conclusions

### 6.1. Conclusions and Policy Implication

This study examined Chinese urban consumers’ own WTP and inferred WTP for low-carbon beef based on the DBDC-CVM and IVMs. The findings implied that the emotional factors of warm glow feelings and protest beliefs and the contextual factor of social norms significantly influenced consumers’ own WTP for low-carbon beef, whereas their inferred WTP was mainly affected by social norms. Their environmental concern had no statistically significant effect on either own WTP or inferred WTP. The inferred WTP for low-carbon beef was RMB 45.29/500 g presenting a premium rate of 13.23%, indicating that urban Chinese consumers generally maintained a willingness to pay a price premium for low-carbon beef.

This study offers several policy implications. First, the research demonstrated that consumers’ own WTP for low-carbon beef was significantly driven by warm glow feelings, particularly the sense of self-affirmation stemming from the specific consumption act. Therefore, a key policy recommendation is that the government should not frame low-carbon beef consumption as an externally imposed environmental obligation, but as a source of personal psychological benefit that allows consumers to evolve into their preferred self. Second, our analysis identified protest beliefs, notably skepticism regarding the payment mechanism, as a key negative determinant of own WTP. Therefore, we propose that the government should mandate transparency regarding the allocation of premium payments and establish accessible channels for consumer feedback and complaints, thereby enhancing consumer trust in the whole system. Third, given the significant impact of social norms, especially proximal peer descriptive norms, on both consumers’ own WTP and inferred WTP, we suggest that the government should prioritize efforts to make low-carbon beef consumption more visible, prevalent, and routine. This can be achieved by, for example, establishing low-carbon consumption role models and showcasing the consumption behaviors of familiar groups within communities to enhance their credibility as social references. The ultimate goal is to transform low-carbon consumption from an individual choice into a collective norm.

### 6.2. Limitations and Future Directions

This study has some limitations. First, it focused on urban consumers in Beijing, which may not fully represent the broader Chinese urban consumer population. Future research should therefore draw on more geographically and socioeconomically diverse samples to better capture the heterogeneity of Chinese urban consumers. Second, although data collected from online and face-to-face were generally homogeneous, a large discrepancy between them may affect the validity of the questionnaires. Future research should be conducted with reasonable sampling methods to test the robustness of our findings. Third, it should be acknowledged that, even with the application of the IVM to address hypothetical bias, hypothetical purchasing scenarios may still introduce limitations. Self-reported behavior may not always accurately reflect actual purchasing patterns. Future studies should incorporate real purchasing data made in virtual purchasing environments to validate the self-reported findings. Last, while our study includes three key social and psychological factors, there may be other important variables that were not captured in this study. Future research could explore more factors to provide a more comprehensive understanding of low-carbon food consumption.

## Figures and Tables

**Table 1:** Demographic characteristics of the survey sample (n = 760).

Variable	Description	Frequency	Percentage
Gender	Male	351	46.18
	Female	409	53.82
Age	18–25 years old	278	36.58
	26–35 years old	315	41.45
	36–45 years old	138	18.16
	46–60 years old	22	2.89
	Above 60 years old	7	0.92
Education	Middle school and below	4	0.53
	High school	31	4.08
	College	40	5.26
	Bachelor’s degree	429	56.45
	Master’s or Doctoral degree	256	33.68
Monthly income *	Under RMB 3000 (Under USD 437.41)	40	5.26
	RMB 3001–5000 (USD 437.41–729.02)	103	13.55
	RMB 5001–8000 (USD 729.02–1166.43)	152	20.00
	RMB 8001–10,000 (USD 1166.43–1458.04)	172	22.63
	RMB 10,001–20,000 (USD 1458.04–2916.08)	183	24.08
	Above RMB 20,000 (Above USD 2916.08)	110	14.48
Having children	Yes	210	27.63
	No	550	72.37
Having religion	Yes	35	4.61
	No	725	95.39
Frequency of eating beef	Never	12	1.58
	Less than 1 time per month	167	21.97
	2–3 times per month	313	41.19
	1–3 times per week	211	27.76
	4–6 times per week	45	5.92
	Every day	12	1.58

*: The exchange rate is 1 USD = 6.8587 RMB, with the reference date being 27 February 2026.

**Table 2:** Demographic variable definitions and descriptive statistics (n = 760).

Variables	Definition	Mean	SD
Gender	1 = Male; 2 = Female	1.54	0.50
Age	1 = 18–25 years old; 2 = 26–35 years old; 3 = 36–45 years old; 4 = 46–60 years old; 5 = more than 60 years old	1.89	0.84
Education	1 = Middle school and below; 2 = High school; 3 = College; 4 = Bachelor’s degree; 5 = Master’s or Doctoral degree	4.19	0.75
Income	Monthly personal income: 1 = Under RMB 3000; 2 = RMB 3001–5000; 3 = RMB 5001–8000; 4 = RMB 8001–10,000; 5 = RMB 10,001–20,000; 6 = Above RMB 20,000	3.90	1.42
Children	1 = Having children; 0 = Otherwise	0.28	0.45
Religion	1 = Having a religion; 0 = Otherwise	0.05	0.21
Frequency	Frequency of eating beef: 1 = Never; 2 = Less than 1 time per month; 3 = 2–3 times per month; 4 = 1–3 times per week; 5 = 4–6 times per week; 6 = Every day	3.19	0.95

**Table 3:** Results of principal component analysis of warm glow feelings, protest beliefs, social norms and environment concern variables (n = 760).

Measure Variables	Mean	SD	Factor Loading
Warm Glow Feelings (WG) (Cronbach’s alpha = 0.878, Variance Explained = 73.32%, KMO = 0.830)			
(1) I would feel good about myself if I decided to take personal action to help reduce climate change. (WG1)	3.65	0.92	0.814
(2) I would feel positive if I contributed to the well-being of humanity and nature. (WG2)	3.85	0.92	0.871
(3) Buying low-carbon foods makes me feel respected. (WG3)	3.69	0.94	0.872
(4) Buying low-carbon foods gives me a pleasant feeling of personal satisfaction. (WG4)	3.63	1.01	0.866
Protest Beliefs (PB) (Cronbach’s alpha = 0.836, Variance Explained = 60.88%, KMO = 0.821)			
(1) I refuse to contribute to carbon reduction in monetary terms. (PB1)	3.17	1.14	0.759
(2) It is unfair for me to pay more money for low-carbon foods. (PB2)	3.16	1.10	0.802
(3) The government should pay to reduce carbon emissions from foods. (PB3)	3.55	1.07	0.851
(4) Those food companies with high carbon emissions should pay for the measures. (PB4)	3.65	1.04	0.819
(5) My money won’t make a difference because most people are not willing to pay for it. (PB5)	3.15	1.10	0.656
Social Norms (SN) (Cronbach’s alpha = 0.775, Variance Explained = 81.70%, KMO = 0.500)			
(1) My family and friends would prefer to pay more for low-carbon foods. (SN1)	3.67	0.88	0.904
(2) People around me generally believe that buying low-carbon foods is more beneficial for the environment. (SN2)	3.67	0.94	0.904
Environmental Concern (EC) (Cronbach’s alpha = 0.802, Variance Explained = 63.13%, KMO = 0.780)			
(1) The current global situation of greenhouse gas emissions is quite severe.	4.14	0.77	0.804
(2) Environmental issues are more critical now than in the past decade.	4.21	0.93	0.801
(3) Climate change has already affected my daily life.	3.74	0.93	0.758
(4) If current trends continue, we will soon suffer a severe environmental disaster.	3.91	0.99	0.814

Note: All items were measured on a 5-point Likert scale (1 = Strongly Disagree, 5 = Strongly Agree).

**Table 4:** Response distribution for the double-bounded dichotomous choice CVM and IVM (n = 760).

Bid Scenario	Bid Value (B_0_, B_H,_ B_L_)	Sample Size (n)	Own Acceptance (Yes) or Rejection (No) of the 1st and 2nd Bids (1st–2nd), n (%)				Inferred Acceptance (Yes) or Rejection (No) of Others of the 1st and 2nd Bids (1st–2nd), n (%)			
			Yes-Yes	Yes-No	No-Yes	No-No	Yes-Yes	Yes-No	No-Yes	No-No
1	(42, 44, 41)	130	72 (55.4)	28 (21.5)	8 (6.2)	22 (16.9)	48 (36.9)	22 (16.9)	27 (20.8)	33 (25.4)
2	(44, 46, 42)	131	47 (35.9)	34 (26.0)	21 (16.0)	29 (22.1)	26 (19.8)	24 (18.3)	34 (26.0)	47 (35.9)
3	(46, 48, 44)	125	42 (33.6)	16 (12.8)	18 (14.4)	49 (39.2)	36 (28.8)	15 (12.0)	24 (19.2)	50 (40.0)
4	(48, 50, 46)	125	37 (29.6)	19 (15.2)	16 (12.8)	53 (42.4)	25 (20.0)	20 (16.0)	18 (14.4)	62 (49.6)
5	(50, 52, 48)	126	44 (34.9)	13 (10.3)	17 (13.5)	52 (41.3)	31 (24.6)	11 (8.7)	18 (14.3)	66 (52.4)
6	(52, 54, 50)	123	35 (28.5)	4 (3.2)	18 (14.6)	66 (53.7)	32 (26.0)	10 (8.2)	17 (13.8)	64 (52.0)

Note: B_0_ is the 1st bid value (RMB/500 g); B_H_ is the high value of the 2nd bid (RMB/500 g); B_L_ is the low value of the 2nd bid (RMB/500 g).

**Table 5:** Estimation results of the double-bounded dichotomous choice model for respondents’ own WTP.

	Model 1		Model 2		Model 3	
	Coef.	Std. Err.	Coef.	Std. Err.	Coef.	Std. Err.
WG			2.822 ***	0.410	2.717 ***	0.398
PB			−0.752 ***	0.279	−0.690 **	0.277
SN			1.629 ***	0.378	1.163 ***	0.371
EC			−0.023	0.283	−0.006	0.279
Gender					−0.688	0.540
Age					0.510	0.341
Education					−0.948 ***	0.362
Income					0.365 *	0.196
Children					1.778 ***	0.635
Religion					0.646	1.303
Frequency					0.919 ***	0.285
Constant	47.012 ***	0.283	46.901 ***	0.264	46.178 ***	2.051
Sample size	760		760		760	
Log-likelihood	−977.823		−863.541		−837.642	
Wald Chi-squared	-		154.78		183.01	
Prob > chi2	-		0.00		0.00	

Note: *, **, and *** denotes 10%, 5%, and 1% level of significance respectively.

**Table 6:** WTP estimation using a double-bounded dichotomous choice model with interaction terms.

	Model 3		Model 4		Model 5	
	Coef.	Std. Err.	Coef.	Std. Err.	Coef.	Std. Err.
WG	2.717 ***	0.398	2.839 ***	0.407	2.708 ***	0.397
PB	−0.690 **	0.277	−0.870 ***	0.298	−0.730 **	0.299
SN	1.163 ***	0.371	1.107 ***	0.369	1.169 ***	0.370
EC	−0.006	0.279	−0.011	0.281	0.018	0.281
WG × PB			0.415	0.289		
WG × SN			0.534 **	0.245		
WG × EC			−0.194	0.263		
PB × SN					−0.034	0.273
PB × EC					0.271	0.266
Gender	−0.688	0.540	−0.642	0.539	−0.670	0.539
Age	0.510	0.341	0.509	0.340	0.505	0.341
Education	−0.948 ***	0.362	−0.870 **	0.364	−0.939 **	0.364
Income	0.365 *	0.196	0.381 *	0.195	0.376 *	0.196
Children	1.778 ***	0.635	1.774 ***	0.634	1.771 ***	0.634
Religion	0.646	1.303	0.506	1.304	0.601	1.301
Frequency	0.919 ***	0.285	0.906 ***	0.283	0.901 ***	0.285
Constant	46.178 ***	2.051	45.538 ***	2.056	46.124 ***	2.053
Sample size	760		760		760	
Log-likelihood	−837.642		−834.672		−837.124	
Wald Chi-squared	183.01		187.2		184.05	
Prob > chi2	0.00		0.00		0.00	

Note: *, **, and *** denotes 10%, 5%, and 1% level of significance respectively.

**Table 7:** WTP estimation using double-bounded dichotomous choice mode with underlying survey items.

	Model 6		Model 7		Model 8	
	Coef.	Std. Err.	Coef.	Std. Err.	Coef.	Std. Err.
WG			2.594 ***	0.395	2.621 ***	0.399
PB	−0.623 **	0.278			−0.697 **	0.277
SN	1.069 ***	0.369	1.173 ***	0.368		
WG1	2.506 ***	0.417				
WG2	0.327	0.434				
WG3	0.517	0.435				
WG4	0.349	0.398				
PB1			−0.802 ***	0.290		
PB2			0.604 **	0.326		
PB3			−0.519	0.376		
PB4			−0.240	0.374		
PB5			0.201	0.278		
SN1					1.592 ***	0.444
SN2					−0.036	0.382
EC	−0.052	0.279	0.029	0.277	−0.025	0.278
Gender	−0.596	0.537	−0.451	0.537	−0.733	0.539
Age	0.464	0.340	0.523	0.338	0.513	0.341
Education	−0.948 ***	0.360	−1.068 ***	0.363	−0.993 ***	0.362
Income	0.349 *	0.195	0.370 *	0.193	0.388 **	0.196
Children	1.764 ***	0.635	1.868 ***	0.631	1.736 ***	0.635
Religion	0.850	1.291	0.535	1.289	0.770	1.306
Frequency	0.880 ***	0.283	0.855 ***	0.284	0.924 ***	0.284
Constant	32.733 ***	2.734	49.173 ***	2.401	40.629 ***	2.713
Sample size	760		760		760	
Log-likelihood	−827.82		−832.243		−834.980	
Wald Chi-squared	191.12		189.72		185.16	
Prob > chi2	-		0.00		0.00	

Note: *, **, and *** denotes 10%, 5%, and 1% level of significance respectively.

**Table 8:** Estimation results of the double-bounded dichotomous choice model for the inferred WTP.

	Model 9		Model 10		Model 11	
	Coef.	Std. Err.	Coef.	Std. Err.	Coef.	Std. Err.
WG			0.774 *	0.400	0.723 *	0.395
PB			−0.362	0.276	−0.379	0.277
SN			2.318 ***	0.404	1.900 ***	0.401
EC			−0.386	0.291	−0.364	0.291
Gender					−0.457	0.562
Age					0.631	0.359
Education					−0.999 ***	0.369
Income					0.246	0.204
Children					0.375	0.662
Religion					1.836	1.322
Frequency					0.939 ***	0.298
Constant	45.392	0.285	45.265 ***	0.281	44.837 ***	2.117
Sample size	760		760		760	
Log-likelihood	−1039.383		−986.911		−970.060	
Wald Chi-squared	-		87.05		110.88	
Prob > chi2	-		0.00		0.00	

Note: * and *** denotes 10% and 1% level of significance respectively.

**Table 9:** Descriptive statistics of own WTP and inferred WTP for low-carbon beef (RMB/500 g).

	Min	Max	Mean	SD	Premium Rate (%)	95% C.I.		Wilcoxon Signed-Rank Test Statistic
						Lower	Upper	
Own WTP	31.55	58.28	47.00	4.66	17.49%	46.66	47.33	18.56 ***
Inferred WTP	35.15	54.66	45.29	3.30	13.23%	45.06	45.53	
WTP GAP	−5.22	6.27	1.70	1.94				

*** denotes 1% level of significance.

## Data Availability

The original contributions presented in the study are included in the article/Supplementary Materials, further inquiries can be directed to the corresponding author.

## Supplementary Materials

The following supporting information can be downloaded at: https://www.mdpi.com/article/10.3390/foods15061023/s1. File S1. Questionnaire.
