# Supplementary material for: Assessing the Impact of Social and Psychological Factors on Consumers’ Willingness to Pay for Low-Carbon Beef: Evidence from Urban China
Source: Foods. 2026 Mar 15;15(6):1023. doi: 10.3390/foods15061023 (PMC13025720; doi:10.3390/foods15061023)
Supplement: Supplementary file 1 [file foods-15-01023-s001.zip › foods-4172160-supplementary.pdf]

Questionnaire[1]

Dear Respondents,

We are conducting research to investigate how social and psychological factors affect consumers' willingness to pay for low-carbon beef. Your information will enable us to understand consumers' choices on sustainable foods and contribute to protect our environment. The responses to this survey will be anonymous and no identifying information will be linked to your responses after you complete the survey. This research went through an ethics approval and is in line with the ethical guidelines and privacy requirements of …. . Should any questions or concerns arise about the survey or the project in general please send an email to …. . Thank you.

Q1. Are you ____?

o Male

o Female

Q2. Within which age group do you fall?

o 18-25 years

o 26-35 years

o 36-45 years

o 46-60 years

o Above 60 years

Q3. What is your highest education level?

o Middle school and below

o High school

o College

o Bachelor's degree

o Master's or Doctoral degree

Q4. What is your average monthly income?

o Under RMB 3000

o RMB 3,001-5,000

o RMB 5,001-8,000

o RMB 8,001-10,000

o RMB 10,001-20,000

[1] Since we have six bidding scenarios, there will be six types of questionnaires. Only one type of them is shown here.

o Above RMB 20,000

Q5. Do you have any religion?

o Yes

o No

Q6. Are there any children (below 12 years old) living in your household?

o Yes

o No

Q7. How frequent does your family eat beef?

o Never

o Less than 1 time per month

o 2-3 times per month

o 1-3 times per week

o 4-6 times per week

o Every day

Q8. Imagine you are shopping in a real supermarket where you need to buy fresh raw beef. The price of conventional beef is RMB 40 /500 g. A new type of beef, known as low-carbon beef, has also appeared in the supermarket. It exhibits no differences in nutrition, taste, or appearance compared with conventional beef, but it generates lower carbon emissions during the production process.

Q8.1 Would you be willing to spend RMB 42 to buy 500 g of low-carbon beef?

o Yes

o No

(1) If "Yes", would you be willing to spend RMB 44 to buy 500 g of low-carbon beef?

o Yes

o No

(2) If "No", would you be willing to spend RMB 41 to buy 500 g of low-carbon beef?

o Yes

o No

Q8.2 Do you think an ordinary person would be willing to spend RMB 42 to buy 500 g of low-carbon beef?

o Yes

o No

(1) If "Yes", do you think an ordinary person would be willing to spend RMB 44 to buy 500 g of low-carbon beef?

o Yes

o No

(2) If “No”, do you think an ordinary person would be willing to spend RMB 41 to buy 500 g of low-carbon beef?

o Yes

o No

Q9. Please state your level of agreement with each of these statements.

	Strongly disagree	Disagree	Neutral	Agree	Strongly agree
The current global situation of greenhouse gas emissions is quite severe.	o	o	o	o	o
Environmental issues are more critical now than in the past decade.	o	o	o	o	o
Climate change has already affected my daily life.	o	o	o	o	o
If current trends continue, we will soon suffer a severe environmental disaster.	o	o	o	o	o

Q10. Please indicate your level of support for these statements.

	Strongly disagree	Disagree	Neutral	Agree	Strongly agree
I would feel good about myself if I decided to take personal action to help reduce climate change.	o	o	o	o	o
I would feel positive if I contribute to the well-being of humanity and nature.	o	o	o	o	o
Buying low-carbon foods makes me feel respected.	o	o	o	o	o

Buying low-carbon foods gives me a pleasant feeling of personal satisfaction.	o	o	o	o	o
My family and friends would prefer to pay more for low-carbon foods.	o	o	o	o	o
People around me generally believe that buying low-carbon foods is more beneficial for the environment.	o	o	o	o	o

Q11. Please state your level of agreement with each of these statements.

	Strongly disagree	Disagree	Neutral	Agree	Strongly agree
I refuse to contribute to carbon reduction in monetary terms.	o	o	o	o	o
It is unfair for me to pay more money for low-carbon foods.	o	o	o	o	o
The government should pay to reduce carbon emissions from foods.	o	o	o	o	o
Those food companies with high carbon emissions should pay for the measures.	o	o	o	o	o
My money won't make a difference because most people are not willing to pay for it.	o	o	o	o	o

This is the end of the questionnaire. Thank you for your cooperation!
